# Dynamic base stations selection method for passive location based on GDOP

**DOI:** 10.1371/journal.pone.0272487

**Published:** 2022-12-12

**Authors:** Sheng Miao, Liang Dong, Jingyu Hou

**Affiliations:** 1 College of Big Data and Intelligence Engineering, Southwest Forestry University Kunming, Yunnan, China; 2 Key Laboratory for the Structure and Evolution of Celestial Objects, Chinese Academy of Sciences Kunming, Yunnan, China; 3 School of Information Technology, Deakin University, Victoria, Australia; Jinan University, China, HONG KONG

## Abstract

For the problem of passive location in mobile cellular network, base stations (BSs) selection can improve positioning accuracy. Through the analysis of base station layout in cellular networks, using Geometric Dilution of Precision (GDOP) as the optimization objective, we propose a Dynamic Base Stations Selection (DBSS) method in a cellular unit. This method enables the system to dynamically select the positioning base station when positioning target in the detection area. DBSS mainly include three steps: nearest base station calculation, layout of base stations analysis, and base station selection based on the target location. We mainly focus on the derivation of four-base station dynamic selection (DBSS4) and five-base station dynamic selection (DBSS5) algorithms. In simulation experiments, DBSS4 algorithm and DBSS5algorithm were compared with the state-of-the-art of BSs selection methods. The results show that our proposed method can achieve the exhaustive search in cellular cells and reduce more than 20% of the GDOP cumulative positioning error compared with the fixed four-base station selection algorithm. Meanwhile, the proposed method is more efficient, requires less running time and floating-point operations (FLOPs) than other comparison algorithm, and is independent of localization algorithms.

## 1 Introduction

With the development of the smartphone and Internet of things (IoT), more and more sensors and smartphones are used to data gathering for location in many areas, such as in healthcare [1] and information safety [2], which have been successfully applied in practice. In communication and IoT, wireless passive location is a very important technology which is widely used in many application areas such as transportation, indoor positioning, robot, and mobile cellular networks etc. [3].

There are usually three ways to improve the accuracy of passive location, The first one is to improve the Time Difference Of Arrival (TDOA) positioning algorithm [4]. The second one is to enhance the transferring power or improve the receiver sensitivity which can improve the signal-to-noise ratio and get a higher precision positioning point [5]. The third way is to select base stations (BS) sensors according to the target position to improve the positioning accuracy [6]. In wireless sensor networks, the problem is sensor selection. I In this paper, a sensor is an antenna installed in a base station, so the BS selection problem is the same as the sensor selection problem.

The BSs selection problem can be expressed as follows, according to the target location, that some base stations are selected as the signal receiving base stations in the detection area to ensure that the optimal is achieved under certain rules. BSs selection for target location is widely used in moving source localization [7], autonomous drones navigation [8], communication and other fields [9]. Generally, BS selection can be regarded as an optimization problem. Joshi and Boyd [10] proposed a heuristic solution to solve the linear sensor selection problem. They defined a Boolean vector as the optimization variable, and used convex relaxation to solve the mean square error (MSE) optimization problem. Chepuri and Leus [11] proposed a sparsity promoting method for sensor selection which aimed to minimize the number of selected sensors. Their proposed constraint was based on the estimation error. There are also relevant studies on location methods in noisy environments, such as Li et al. [8] formulated an optimization problem in which the trace of the Cramér-Rao lower bound (CRLB) was minimized, and used convex relaxation to solve it.

Recently, Zhao et al. [3] studied the multi-objective optimization problem based on the single objective optimization problem. They proposed a polyblock outer approximation (POA) algorithm to solve the single-objective optimization problem and in turn to find the global optimal solution. Meanwhile, they also proposed two suboptimal algorithms, named POA-based accelerated cutting (POA-AC) algorithm and POA-based monotonic cutting (POA-MC) algorithm, which can achieve the exhaustive searching. Dai et al. [9] proposed an optimal sensor selection method using a Boolean vector and the mean square error (MSE) was adopted as an optimization target. Their sensor selection method was dependent on the location method it used, therefore, for different location algorithms the selection results were different.

**Fig 1:**
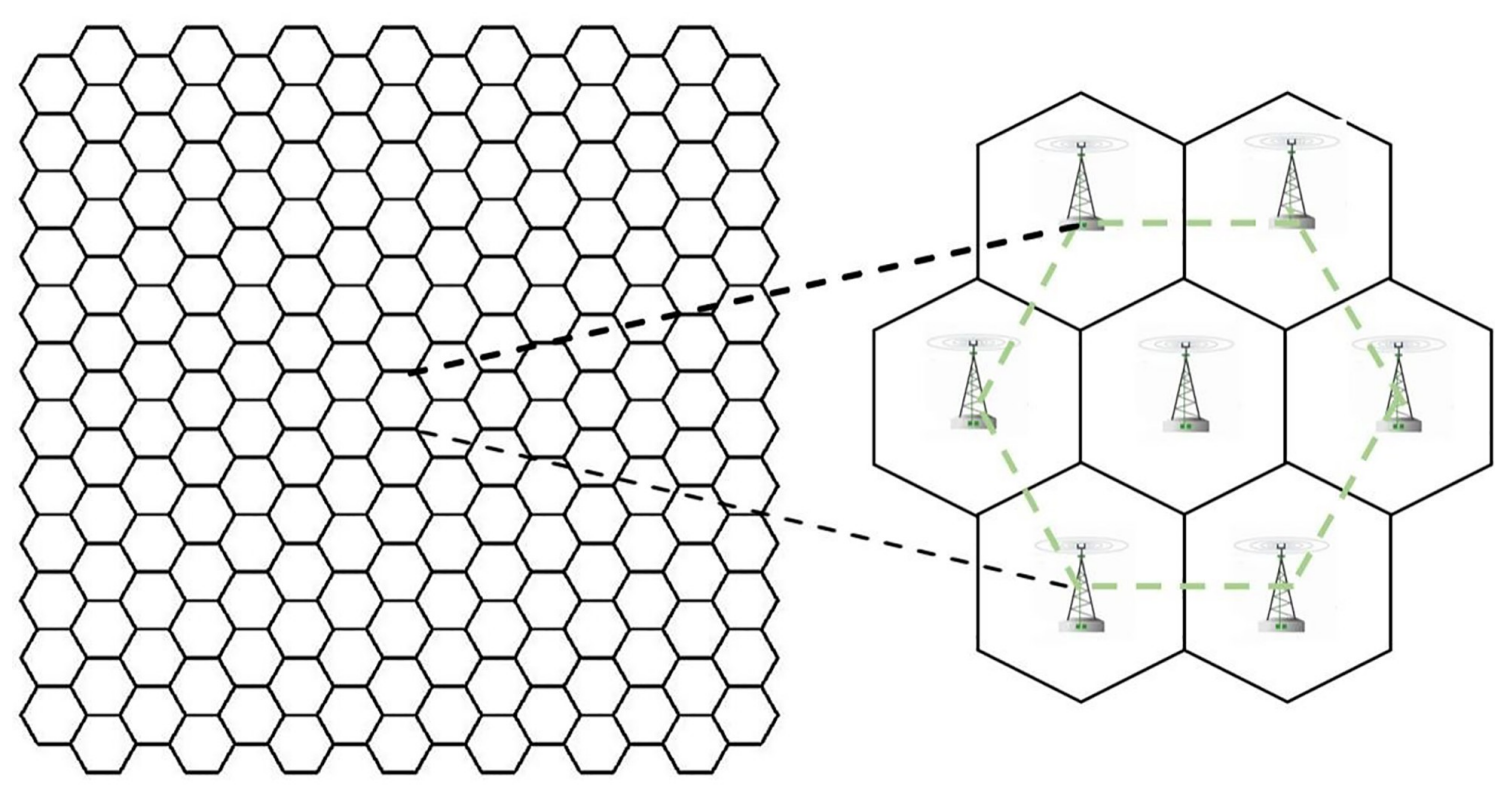
Architecture of conventional cellular networks.

The existing sensor selection algorithms are universal and subject to various constraints, and the calculation is complex [6]. So, the main challenge of BSs selection is that the algorithm is required to be concise and effective, which can quickly update according to the target location, and is independent of the positioning algorithm. In this paper, we mainly focus on the base station selection problem for specific scenarios of mobile cellular networks. In mobile communication networks, passive location of an emitter in a monitoring area is one of the most important applications, because the architecture of mobile cellular networks has been in use for many years and has played an important role in communication, location, detection and other fields [12]. With the development of mobile communication technologies from 2G to 5G, there have been some adjustments and improvements to the technology and structure of cellular networks, but the main architecture is still the same [13–17]. In the architecture of conventional cellular networks, each base station (BS) employs 100 to 120 directional antennas to cover a regular hexagon area. The whole area of a cellular network is covered by a combination of such hexagons. Each base station is surrounded by six base stations forming a regular hexagon which is called a cellular networks unit (cellular cell for short), and the central base station is called main station (MS). The distance between each base station is fixed, which is called cell radius (R). The structure of cellular base stations is shown in Fig 1 [18].

In this paper, we analyze the characteristics of base station layout in cellular network, using Geometric Dilution of Precision (GDOP) as the optimization objective, propose a succinct and high-efficient base station selection method in mobile cellular networks. Our method can select the monitoring base station according to any target location in the monitoring area to minimize the GDOP value. At the same time, the algorithm is simple and efficient, with less computation, which is convenient for real-time dynamic monitoring of targets.

In order to make the algorithm more practical, we provide two dynamic base station selection (DBSS) algorithms for selecting four base stations and five base stations, which are denoted as DBSS4 and DBSS5 respectively. In experiments simulation, we used the running time and floating points (FLOPs) to measure the complexity of the works and comparison algorithms. The results of the experiments verified the effectiveness of our algorithm.

## 2 Methodology

If a base station receives a signal in the detection area of a carrier cellular network, it means that there is a transmitter in the monitoring area. We can use the base stations around the transmitter for initial positioning. One method is to arbitrarily select three base stations whose received signal strength exceeds a threshold for positioning, the selected stations are called an initial base station array.

According to the initial location of the target, the location base station in the cellular network can be re-selected to obtain a higher positioning accuracy. In order to select BSs more reasonably, we analyzed the TDOA algorithm and noticed the factors that affect the positioning accuracy (in terms of GDOP) were the base station measurement error and the target position measurement error. Further analysis showed that the measurement errors in TDOA were determined by the time delay error, which was related to the signal-to-noise ratio (SNR) of a received signal. The SNR of a received signal is related to the attenuation of electromagnetic wave propagation. Therefore, reducing the propagation distance can improve the SNR and reduce the measurement error.

Based on the above analysis, the selection of base stations needs to consider the layout mode to reduce the distance and variance between base stations, while the relative position between the target and each base station needs to be considered as well. Accordingly, our BSs selection method could be divided into three steps: locating the nearest base station, selecting the layout of base stations, and selecting/finalizing the definition of BS array based on the target area.

**Fig 2:**
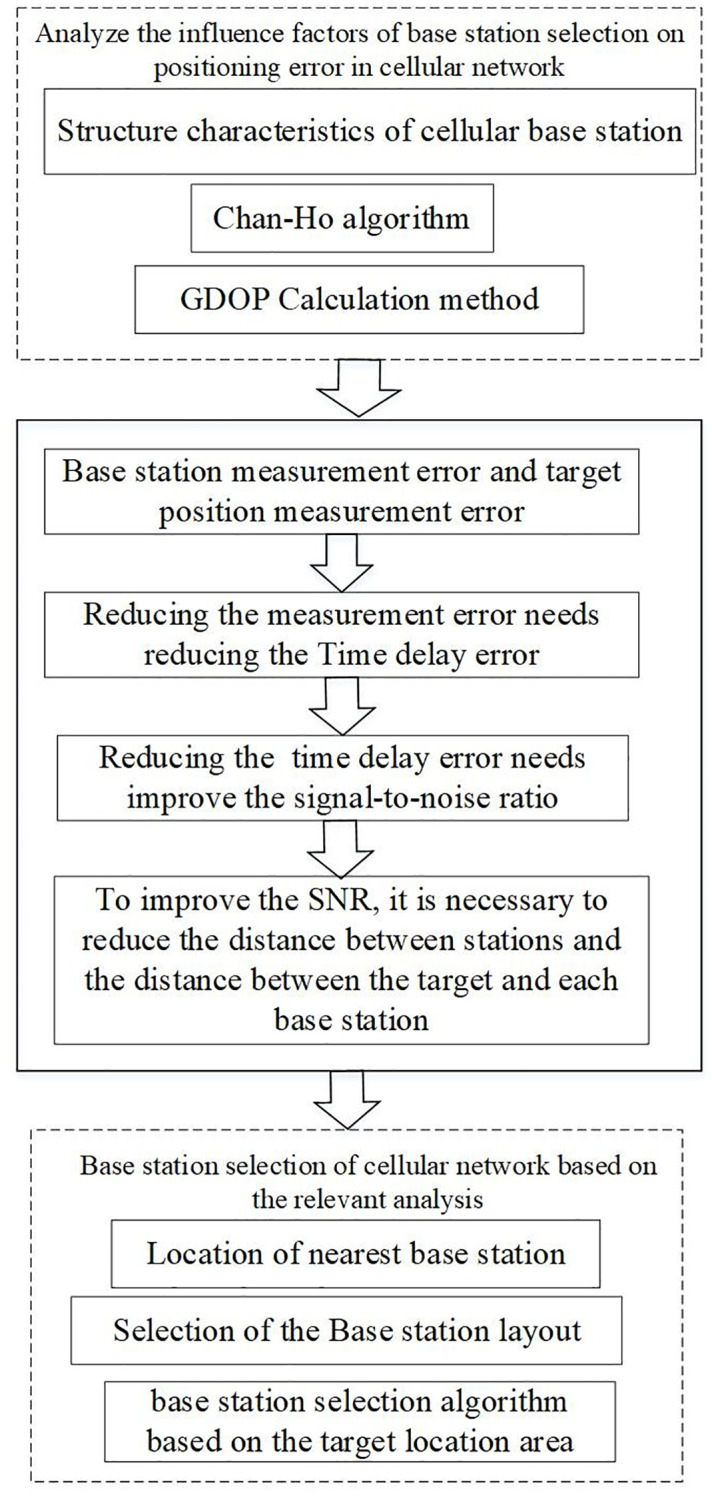
Analysis flow chart of base station selection algorithms in mobile cellular networks.

The flow chart of our analysis on the algorithms of base station selection in mobile cellular networks is shown in Fig 2. In this section, we divided two parts, section 2.1 introduce the background, include Chan-Ho algorithm, GDOP and time delay error analysis. Section 2.2 introduce our DBSS algorithm step by step.

### 2.1 Theoretical background

#### 2.1.1 Chan-Ho algorithm

**Fig 3:**
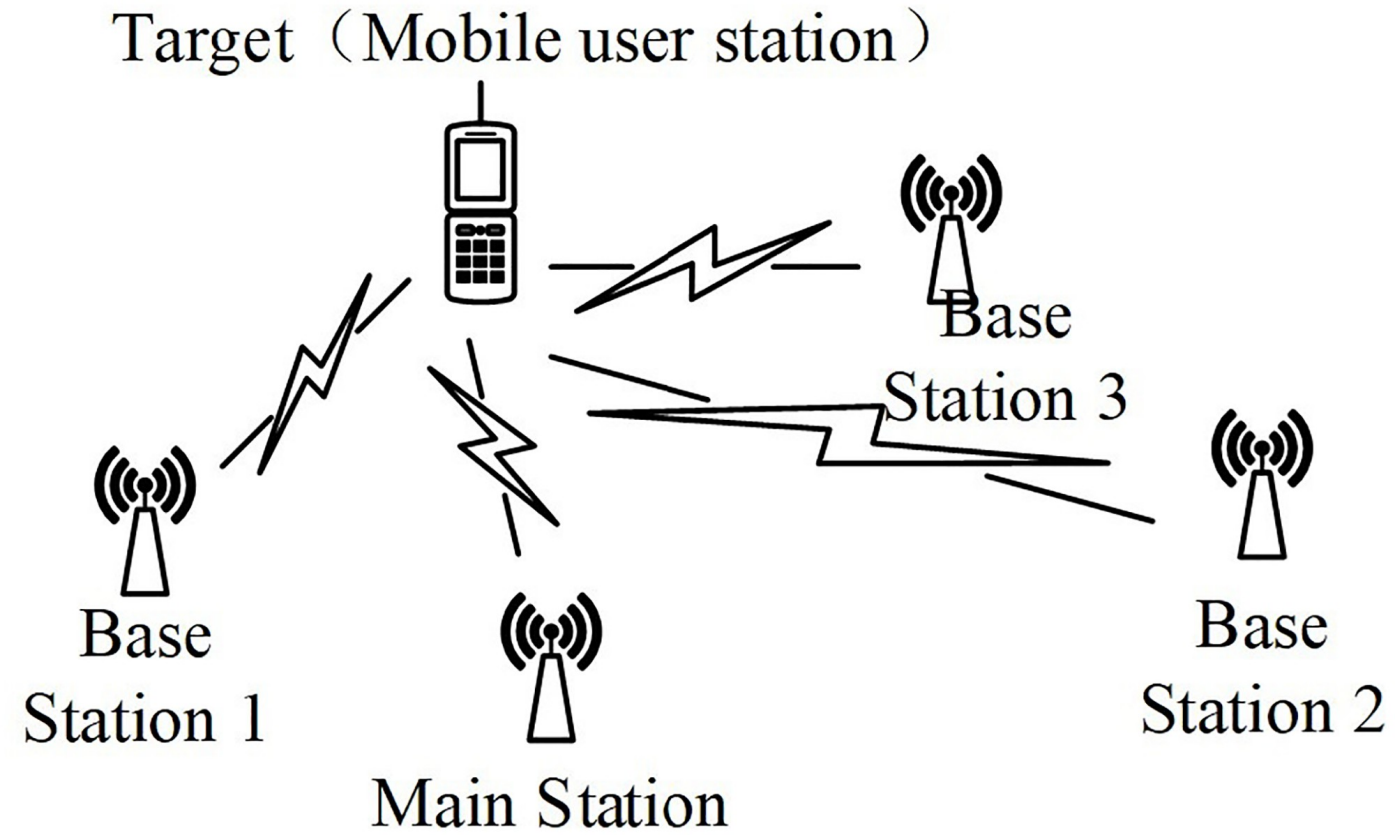
Schematic diagram of TDOA positioning system.

The TDOA location system consists of a main station and N auxiliary base stations. Three stations can locate two dimensional coordinates of a target, while four base stations can locate three dimensional coordinates of a target [19, 20]. As shown in Fig 3, the more base stations we have, the higher positioning accuracy we can achieve.

Chan’s location algorithm [21] is the most widely used technique duo to its calculation simplicity and reliable positioning accuracy. But finding the solution is not easy as the equations in the algorithm are nonlinear. There has been a lot of work to improve Chan’s algorithm, such as the Chan-Ho algorithm [22] which gives an alternative solution to the TDOA hyperbolic equations. Here we use the Chan-Ho algorithm as our location method.

Let the coordinates of base station locations be *r*_*i*_ = (*X*_*i*_, *Y*_*i*_, *Z*_*i*_), *i* = 0, 1, 2, 3. (*X*_0_, *Y*_0_, *Z*_0_) is for the main station, others are for the auxiliary base stations. *r* = (*X*, *Y*, *Z*) is for the target location,

Set *d*_*i*_ be the distance from the target to each base station.

$$\begin{array}{c} d_{i}=\sqrt{{\left(X-X_{i}\right)}^{2}+{\left(Y-Y_{i}\right)}^{2}+{\left(Z-Z_{i}\right)}^{2}} \end{array}$$ (1)

Set

$$\begin{array}{c} \begin{array}{c} R_{0}=\sqrt{X_{0}^{2}+Y_{0}^{2}+Z_{0}^{2}}\\ R_{i}=\sqrt{X_{i}^{2}+Y_{i}^{2}+Z_{i}^{2}} \end{array} \end{array}$$ (2)

*d*_*i*_ will then be:

$$\begin{array}{c} d_{i}=\sqrt{R_{i}^{2}+R_{0}^{2}-2X_{i}X-2Y_{i}Y-2Z_{i}Z} \end{array}$$ (3)

Set *d*_*i*_0 be the distance difference between the ith base station and the main base station:

$$\begin{array}{c} d_{i0}=d_{i}-d_{0}=c\tau_{i0} \end{array}$$ (4)

where *c* is the light speed, and *τ*_*i*_0 is the time delay of receiving signals from the ith base stations to the main station. From Eqs (1) and (3), we can get the following expression:

$$\begin{array}{c} 2{\left(x-x_{i}\right)}^{2}+2{\left(y-y_{i}\right)}^{2}+2{\left(z-Z_{i}\right)}^{2}-2d_{i0}r_{10}=d_{i0}^{2}+R_{0}^{2}-R_{i}^{2} \end{array}$$ (5)

where *r*_10_ = *d*_0_ = |*r*−*r*_0_|, which represents the distance from the target to the main station. Eq (5) can be presented in a matrix format:

$$\begin{array}{c} e+r_{10}C=Er \end{array}$$ (6)

where

$$\begin{array}{c} e=[\begin{array}{c} d_{10}+R_{0}^{2}-R_{1}^{2}\\ \cdots \\ d_{i}^{2}+R_{0}^{2}-R_{i}^{2} \end{array}] \end{array}$$ (7)

$$\begin{array}{c} E=2[r_{0}-r_{1}\;r_{0}-r_{2}\;\cdots \;r_{0}-r_{i}] \end{array}$$ (8)

$$\begin{array}{ccc} C=2[d_{10} & d_{20} & \cdots ] \end{array}$$ (9)

From Eq (6), we get the solution as follow:

$$\begin{array}{c} r={\left(E^{T}E\right)}^{-1}E^{T}(e+r_{10}C) \end{array}$$ (10)

For *r*_10_, we have:

$$\begin{array}{c} r_{10}^{2}={\left(r_{1}-r\right)}^{T}(r_{1}-r) \end{array}$$ (11)

From Eqs (6) and (11), we have:

$$\begin{array}{c} a_{1}r_{10}^{2}+e_{1}r_{10}+c_{1}=0 \end{array}$$ (12)

where:

$$\begin{array}{c} \begin{array}{c} a_{1}=1-C^{T}AC\\ c_{1}=2C^{T}(E{\left(E^{T}E\right)}^{-1})r_{1}-2C^{T}Ac\\ c_{1}=2r_{1}^{T}{\left(B^{T}B\right)}^{-1}B^{T}e-r_{1}^{T}r_{1}-e^{T}Ae \end{array} \end{array}$$ (13)

and

$$\begin{array}{c} A=E{\left(E^{T}E\right)}^{-1}{\left(E^{T}E\right)}^{-1}E^{T} \end{array}$$ (14)

It is clear that the roots of Eq (12) are:

$$\begin{array}{c} {\hat{r}}_{10}=\frac{-e_{1}\pm \sqrt{e_{1}^{2}-4a_{1}c_{1}}}{2a_{1}} \end{array}$$ (15)

For these two solutions of the equation, we discard the negative root and bring the positive root into the formula (10), and then the estimation of the target is obtained as follow:

$$\begin{array}{c} \hat{r}={\left(E^{T}E\right)}^{-1}E^{T}(e+r_{10}C) \end{array}$$ (16)

#### 2.1.2 Geometric dilution of precision analysis

In passive location, Geometric Dilution of Precision (GDOP) is one of the most important standards to measure the accuracy of positioning. The GDOP value represents the positioning error of each point in the monitoring area [19, 23, 24]. It is defined as follow:

$$\begin{array}{c} GDOP=\sqrt{\sigma_{X}^{2}+\sigma_{y}^{2}+\sigma_{z}^{2}} \end{array}$$ (17)

where $\sigma_{x}^{2}$, $\sigma_{y}^{2}$, $\sigma_{z}^{2}$ are the variances of *X* = (*x*, *y*, *z*) respectively. Then, we need to calculate the covariance matrix of *dX*. Set:

$$\begin{array}{c} \Delta r_{i}=d_{i}-d_{0} \end{array}$$ (18)

and perform a differential operation on both sides of Eq (18), we have:

$$\begin{array}{c} d(\Delta r_{i})=(c_{ix}-c_{0x})\text{dx}+(c_{iy}-c_{0y})\text{dy}+(c_{iz}-c_{0z})\text{dz}+(k_{i}-k_{0}) \end{array}$$ (19)

where $c_{jx}=\frac{\partial r_{j}}{\partial x}=\frac{x-x_{j}}{r_{j}}$, $c_{jy}=\frac{\partial r_{j}}{\partial y}=\frac{y-y_{j}}{r_{j}}$, $c_{jz}=\frac{\partial r_{j}}{\partial z}=\frac{z-z_{j}}{r_{j}}$.

Set *k*_*j*_ = *c*_*jx*_d*x*_*j*_ + *c*_*jy*_d*y*_*j*_+ *c*_*jz*_d*z*_*j*_, (*j* = 0, … *N*)

The matrix form of expression (19) is

$$\begin{array}{c} dR=CdX+dS \end{array}$$ (20)

Where

$$\begin{array}{c} C=[\begin{array}{ccc} c_{1x}-c_{0x} & c_{1y}-c_{0y} & c_{1z}-c_{0z}\\ \vdots & \vdots & \vdots \\ c_{nx}-c_{0x} & c_{ny}-c_{0x} & c_{nz}-c_{0x} \end{array}] \end{array}$$ (21)

$$\begin{array}{c} dR={\left[d\left(\Delta r_{10}\right),d\left(\Delta r_{20}\right),...d\left(\Delta r_{i0}\right)\right]}^{T}=c\left[d\tau_{10},d\tau_{20},...d\tau_{i0}\right] \end{array}$$ (22)

$$\begin{array}{c} dS={\left[k_{1}-k_{0},k_{2}-k_{0},...k_{i}-k_{0}\right]}^{T},i=1,2,...N \end{array}$$ (23)

$$\begin{array}{c} dX={\left[dx,dy,dz\right]}^{T} \end{array}$$ (24)

*τ*_*i*0_ is the time delay of receiving signals from the ith base stations to the main station The estimated value of *dX* can be obtained by using a pseudo inverse matrix:

$$\begin{array}{c} d\hat{X}={\left(C^{T}C\right)}^{-1}C^{T}\left(dR-dS\right) \end{array}$$ (25)

Set

$$\begin{array}{c} B={\left(C^{T}C\right)}^{-1}C^{T}={\left[b_{ij}\right]}_{3*N} \end{array}$$ (26)

Then

$$\begin{array}{c} P=E\left[d\hat{X}\cdot d{\hat{X}}^{T}\right]=B\left\{E\left[dR\cdot dR^{T}\right]+E\left[dS\cdot dS^{T}\right]\right\}B^{T} \end{array}$$ (27)

For simplicity, set

$$\begin{array}{c} E\left[dR\cdot dR^{T}\right]+E\left[dS\cdot dS^{T}\right]={\left[\sigma_{ij}^{2}\right]}_{N\times N} \end{array}$$ (28)

The GDOP then can be expressed as:

$$\begin{array}{c} GDOP=\sqrt{\sigma_{x}^{2}+\sigma_{y}^{2}+\sigma_{z}^{2}}=\sqrt{\sum_{i=1}^{N}\sum_{j=1}^{N}\left(b_{1i}b_{1j}+b_{2i}b_{2j}+b_{3i}b_{3j}\right)\sigma_{ij}^{2}} \end{array}$$ (29)

Where $\sigma_{ij}^{2}$ is the position measurement error between the ith and jth base stations. More detailed derivations can be found in the references.

From expression (28), we can see that GDOP is mainly related to the measurement error between base stations (dS) and the target measurement error (dX), which is calculated by the measurement errors $\left(\sigma_{x}^{2}\sigma_{y}^{2}\sigma_{z}^{2}\right)$ on *x*, *y*, and *z* axes. Formula (29) shows that in TDOA location, the measurement error is determined by the time delay error *σ*_*ij*_. Furthermore, according to expression (4), the position difference equals to the time difference multiplied by the light speed. Therefore, the position measurement error $\sigma_{ij}^{2}$ between two base stations is proportional to the delay error $\sigma_{\tau }^{2}$. So it is necessary to discuss the calculation of time delay error in TDOA.

#### 2.1.3 Time delay error analysis

The measurement error of TDOA is mainly related to the time delay parameter (*τ*). Based on the analysis of the delay error with the Cramer-Rao bound, the factors influencing the delay error can be obtained [25, 26]. Cramer-rao lower bound of time delay error *σ*_*τ*_ can be expressed by Coherent signal [24]:

$$\begin{array}{c} \sigma_{\tau }^{2}\geq \frac{1}{2T\int_{0}^{\infty }{\left(2\pi f\right)}^{2}\frac{\left|C(f)\right|}{1-{\left|C(f)\right|}^{2}}df} \end{array}$$ (30)

where *C*(*f*) is the coherence function which is defined as:

$$\begin{array}{c} {\left|C(f)\right|}^{2}=\frac{P_{ss}^{2}\left(f\right)}{{\left|P_{ss}\left(f\right)+P_{nn}\left(f\right)\right|}^{2}} \end{array}$$ (31)

Here, *P*_*ss*_(*f*) is the signal autocorrelation spectrum and *p*_*nn*_(*f*) is the noise autocorrelation spectrum. They are defined as:

$$\begin{array}{c} P_{ss}\left(f\right)=\int_{-\infty }^{+\infty }R_{SS}(\tau )e^{j2\pi f\tau }d\tau \end{array}$$ (32)

$$\begin{array}{c} P_{nn}\left(f\right)=\int_{-\infty }^{+\infty }R_{nn}(\tau )e^{j2\pi f\tau }d\tau \end{array}$$ (33)

Regarding expression (27), we have:

$$\begin{array}{c} \frac{\left|C(f)\right|}{1-{\left|C(f)\right|}^{2}}=\frac{\frac{P_{ss}^{2}\left(f\right)}{{\left|P_{ss}\left(f\right)+p_{nn}\left(f\right)\right|}^{2}}}{1-\frac{P_{ss}^{2}\left(f\right)}{{\left|P_{ss}\left(f\right)+p_{nn}\left(f\right)\right|}^{2}}}\approx {\left(SNR\right)}^{2} \end{array}$$ (34)

where SNR is the received signal-to-noise ratio defined as:

$$\begin{array}{c} SNR=\frac{P_{SS}^{2}\left(f\right)}{P_{nn}^{2}\left(f\right)} \end{array}$$ (35)

In order to effectively identify correlation peaks, we generally require that the signal-to-noise ratio (SNR) is greater than 10dB. Under this condition, we assume SNR>>1, then *σ*_*τ*_ can be approximately expressed as

$$\begin{array}{c} \sigma_{\tau }\approx \sqrt{\frac{3}{4\pi^{2}T}}\frac{1}{\sqrt{SNR}}\frac{1}{\sqrt{f_{2}^{2}-f_{1}^{2}}} \end{array}$$ (36)

Then, the error variance of the received signal is directly proportional to the reciprocal of the SNR of the received signal, i.e.,

$$\begin{array}{c} \sigma_{\tau }^{2}\propto \frac{1}{SNR} \end{array}$$ (37)

SNR of a received signal in a signal receiver is affected by two factors: one is receiver SNR, another is transmission SNR. The first one is determined by the receiver performance, therefore we consider it as a fixed value.

For the transmission SNR, it is determined by the loss of electromagnetic wave propagation, which is a problem of Non-line-of-sight error mitigation, and it was analyzed in detail for TDOA systems [27]. Here, for simplicity, we only consider an ideal situation where the transmission loss of electromagnetic wave is approximately expressed as follow [28]:

$$\begin{array}{c} \beta (dB)=32.44+20\text{lg}L(km)+20\text{lg}F(MHz) \end{array}$$ (38)

where L is the transmission distance in terms of kilometer (km), and F is the transmission frequency in terms of megahertz (MHz).

It can be estimated from the above formula that the transmission SNR will decrease about 6dB for every doubling of the transmission distance of electromagnetic wave. Therefore, we should try to reduce the distance between the target point and each base station, as well as the distance between each base station when we choose the BSs.

### 2.2 Dynamic Base Stations Selection (DBSS) method

#### 2.2.1 Nearset base station selection

In the cellular network architecture shown in Fig 1, the base stations are arranged in the form of regular hexagon. Therefore, the selection of BS must take into account the cellular network architecture [12]. We need to locate the most suitable cellular network cell according to the target location. The nearest base station method was used to seek the most suitable cell unit in a cellular network.

For convenience, here we set z = 0 and Z-axis is not considered, then the cellular origin location is set as (*x*_0_, *y*_0_)=(0,0), and the target location is (*x*, *y*). Set the distance between base stations (cell radius R) as 1. Then the location point of each base station can be summarized as follows with coordinates.

**Fig 4:**
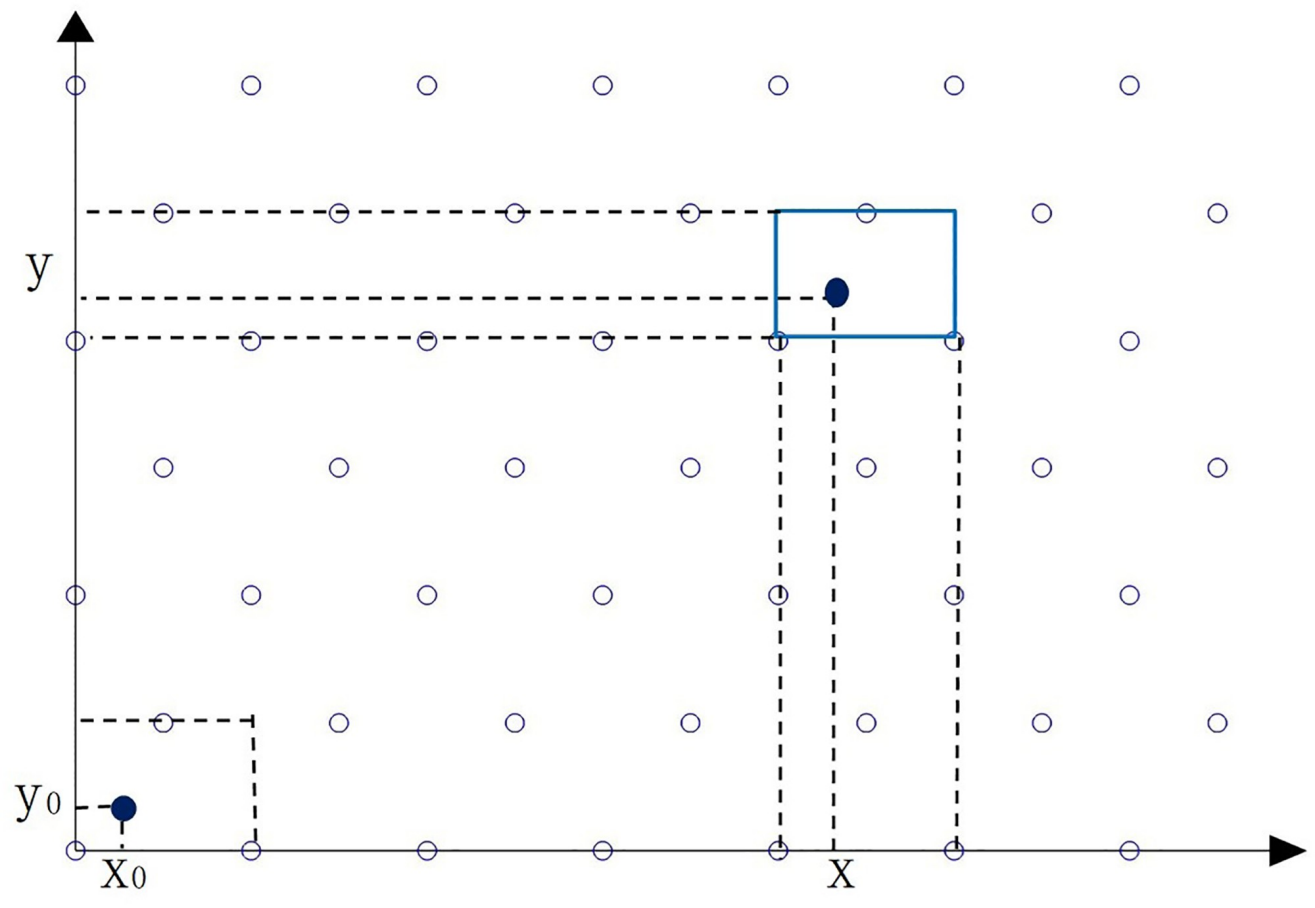
Coordinate arrangements of cellular base stations and the target location.

The Y-axis positions are marked as $\left\{0,\sqrt{3},2\sqrt{3},...,n\sqrt{3}\right\}$, while the X-axis positions are a regular arrangement of {, 1, 2, 3…, *n*}. For the Y-axis positions marked as $\left\{\sqrt{3}/2,3\sqrt{3}/2,\ldots ,\left(2n-1\right)\sqrt{3}/2\right\}$, the X-axis position are a regular arrangement of $\left\{1/2,3/2,\ldots ,\left(2n-1\right)/2\right\}$ According to the arrangement rule of cellular base stations, the whole area can be divided into multiple rectangular areas, each of which is with 1 in length and $\sqrt{3}/2$ in height. Each rectangular area has three base stations, which are respectively located in the center area which is identified by the two left and right vertices and the upper edge of the rectangle, as shown in Fig 4.

In cellular networks, if the monitoring signal of a base station exceeds a defined threshold level, there will be a target source nearby. At this time, the base station can be used as the main base station, and 2–3 auxiliary base stations can be randomly selected to form the initial station array to locate the target source as the target initial location.

Set the target initial location be (*x*, *y*), the method for searching the nearest base station location is described as follows. Firstly, the abscissa and ordinate of the target location are re-represented as follows:

$$\begin{array}{c} x=n+x_{0} \end{array}$$ (39)

$$\begin{array}{c} y=\frac{\sqrt{3}}{2}m+y_{0} \end{array}$$ (40)

where *m* and *n* are positive integers, which are transformed by integral division, the remainder (*x*_0_, *y*_0_) was limited in the rectangular area with length 1, width $\sqrt{3}/2$.

Secondly, calculate the Euclidean distance from the target to the three base stations whose locations are (0,0), (0,1) and (1/2, $\sqrt{3}/2$) respectively. Find the base station with the shortest distance.

Thirdly: Add the base station location coordinate $\left(n,\frac{\sqrt{3}}{2}m\right)$, and then the location of the nearest base station can be obtained. Take the nearest base station as the central base station to build a cell unit, and the target will be limited to the central area of the cell unit, as shown in the gray part of Fig 6 (a). There are seven base stations in the regular hexagon area of the cellular cell, base stations will be selected from them.

Take the selection of four base stations as an example, in addition to the central base station, three base stations need to be selected from six surrounding stations. To choose three out of the six base stations, there are $C_{6}^{3}=20$ ways to take. Theoretically, by calculating the GDOP values of different site combinations and selecting the base station combination with the minimum value, the corresponding optimal solution can be obtained.

Considering that there are too many ways of selecting base stations (20 options), and the calculation of GDOP value is too complicated while the attenuation of electromagnetic wave is uncertain, it is necessary to use a simplified base station selection method in cellular networks.

From the definition of GDOP in expression 17 and 20, the main factor that affects the positioning accuracy is the measurement error, which is composed of two parts: *dR* is the measurement error from the target to each station and *dX* is the measurement error of each station. The measurement error is proportional to the measuring distance because the farther the distance is in expression (36), the greater the attenuation of electromagnetic wave and the measurement error will be. Therefore, we need to change the way of selecting BSs from selecting the minimum distance sum to choosing the station layout and positioning BSs.

#### 2.2.2 Selection of base station layout mode

**Fig 5:**
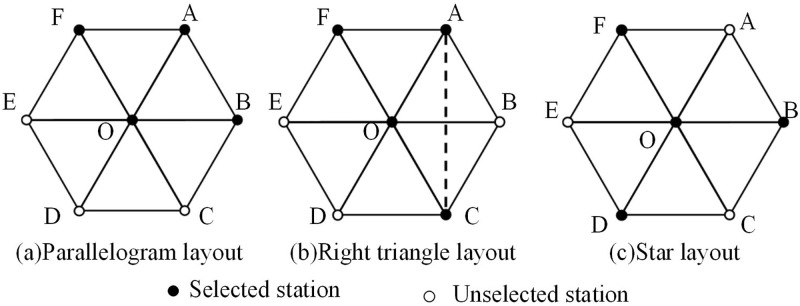
Three kinds of layout in cellular networks. (a) Parallelogram layout, (b) Right triangle layout, (c) Star layout. • Selected station, ○ Unselected station.

Four base stations can form a variety of distribution layout [19]. However, in the architecture of cellular cell, there are only three station layouts to choose, which are shown in Fig 5. One is a parallelogram layout shown in Fig 5(a), selecting F, A, B and O stations. Another is a right triangle layout shown in Fig 5(b), selecting A, C, O and F stations. The third one is a star layout shown in Fig 5(c), selecting F, B, D and O stations.

In order to optimize the GDOP, it is necessary to reduce the distance between base stations and minimize the maximum distance. The distance and variance between base stations are used as indicators to measure the advantages and disadvantages of each station layout. For the Parallelogram layout (FOBA), the sum of distance and variance can be calculate as follow:

$$Dis=|OF|+|OA|+|OB|+|AF|+|AB|+|FB|=5+\sqrt{3},\text{Variance}=0.0886.$$

For the right triangle layout (ACOF), the sum of distance and variance can be calculated as:

$$Dis=|OF|+|OA|+|OC|+|AF|+|AC|+|FC|=6+\sqrt{3},\text{Variance}=0.2336.$$

For the star layout (FOBD), the sum of distance and variance can be calculated as:

$$Dis=|OF|+|OD|+|OB|+|FB|+|BD|+|FD|=3+3\sqrt{3},\text{Variance}=0.1607.$$

Compared with other two layouts, the parallelogram layout is better in terms of distance sum and variance. Therefore, we choose the parallelogram layout in our method.

#### 2.2.3 Base station selection

**Fig 6:**
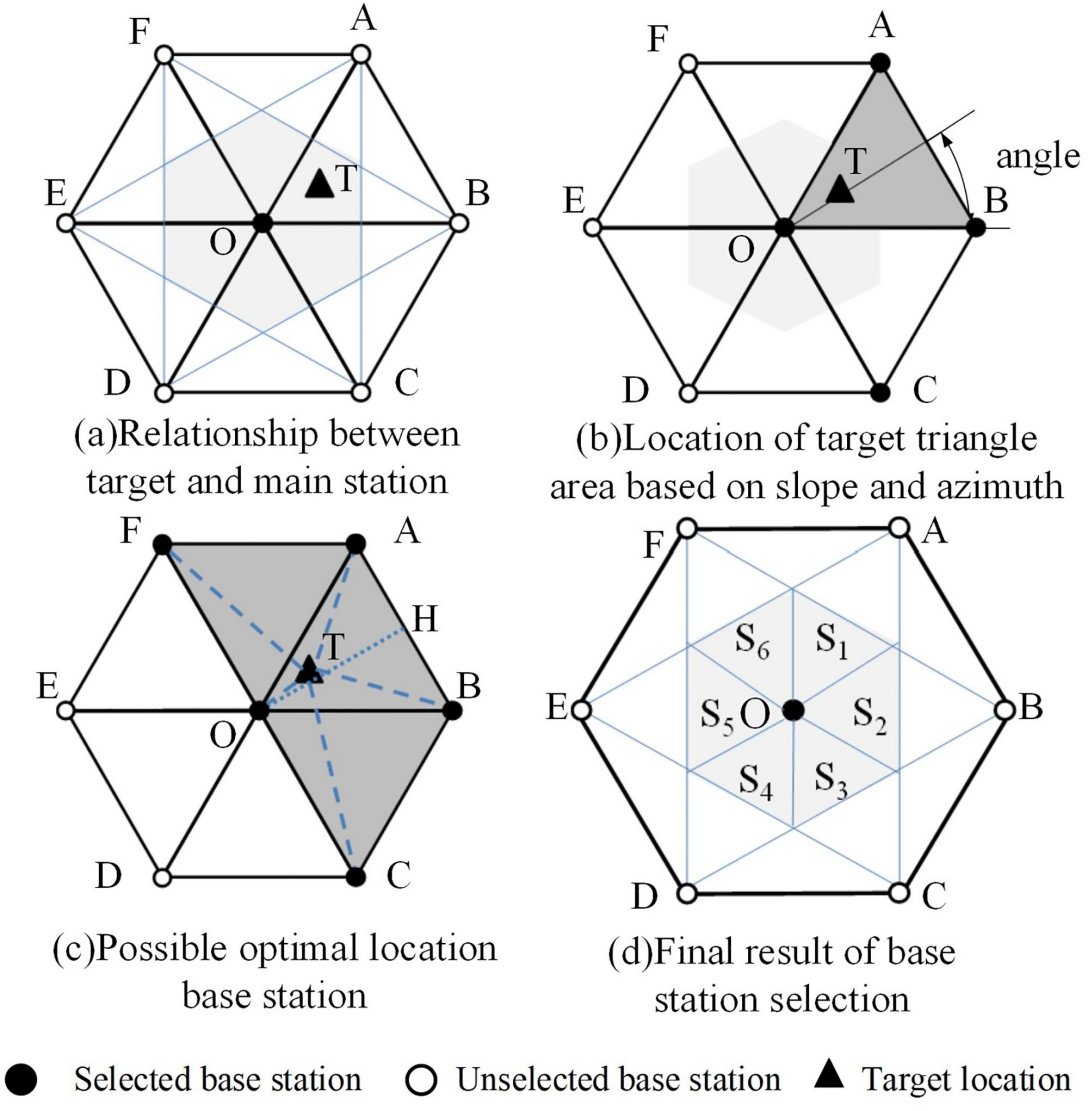
Base station selection based on nearest neighbor. (a) Relationship between target and main station, (b) Location of target triangle area based on slope and azimuth, (c) Possible optimal location base station, (d) Final result of base station selection. • Selected base station, ○ Unselected base station, ▲ Target location.

We have discussed the selection of parallelogram layout in section E. Actually, six groups of parallelogram layout modes can be formed in a cellular cell. Take Fig 6(a) as an example, they are ▫*FOBA*, ▫*AOCB*, ▫*BODC*, ▫*COED*, ▫*DOFE* and ▫▫*OAF*. Selecting one from them is based on the distance from the target to each station.

Firstly, the quadrant of the locating point relative to the nearest point is determined by the values of *y*−*y*_1_ and *x*−*x*_1_, i.e., whether they are positive or negative. Then, the triangle area of the positioning point is determined by calculating the slope: $k=\frac{y-y_{0}}{x-x_{0}}$, By calculating the slope, we can determine the triangle area where the location point is determined by the slope sum, the relative position of the estimated point and the nearest base station. We can estimate the tilt angle and determine which base station is most likely to be selected as the location base station.

For example, suppose that the target location which is relative to the location point (*x*_0_, *y*_0_) is (*x*, *y*), the inclination angle is (0, *π*/3), and the locations of other two base stations are (1,0) and (0.5, $\sqrt{3}/2$) respectively, as shown in Fig 6(b). We find out two base stations (F and C) adjacent to this triangle area, as shown in Fig 6(c). This can exclude the base station on the other side of the hexagon, as there are only two ways to choose a parallelogram layout.

For the target T, its cellular cell has been determined, and the triangle area can also be determined by the slope described in section F. The last step is to choose the parallelogram base station. Take Fig 6(c) as an example, target T has been determined in *ΔOAB*, the fourth station is selected from either station F or C. It can be determined simply by comparing the distance:

If |*TC*| ≥ |*TF*|, select F; else select C.

The property of equilateral triangle center line can be used to judge the distance between the target location and the base station. As shown in Fig 6(c), set OH as the triangle center line, if the target is within ΔOHA, select F, if it is in ΔOHB, select base station C. According to the above rules, the rules of dynamic base station selection in different areas of cellular networks can be summarized in Fig 6(d). The DBSS4 method is as follows:

*S*_1_ → ▫*FABO*, *S*_2_ → ▫*ABCO*, *S*_3_ → ▫*BCDO*, *S*_4_→_▫_ *CDEO*, *S*_5_ → ▫*DEFO*, *S*_6_ → ▫*EFAO* where ▫ represents the parallelogram followed by letters that represent the vertices of the base stations.

In TDOA location, the more base stations are used, the higher the location accuracy is, but the higher the system overhead is. Therefore, we need to make a balance between positioning accuracy and system efficiency. Through the simulation analysis (part III), the positioning accuracy of five base stations is better than that of four base stations, but more base stations (such as six base stations or their base stations) cannot improve the positioning accuracy effectively. Therefore, the dynamic selection method of five BSs is also given here.

**Fig 7:**
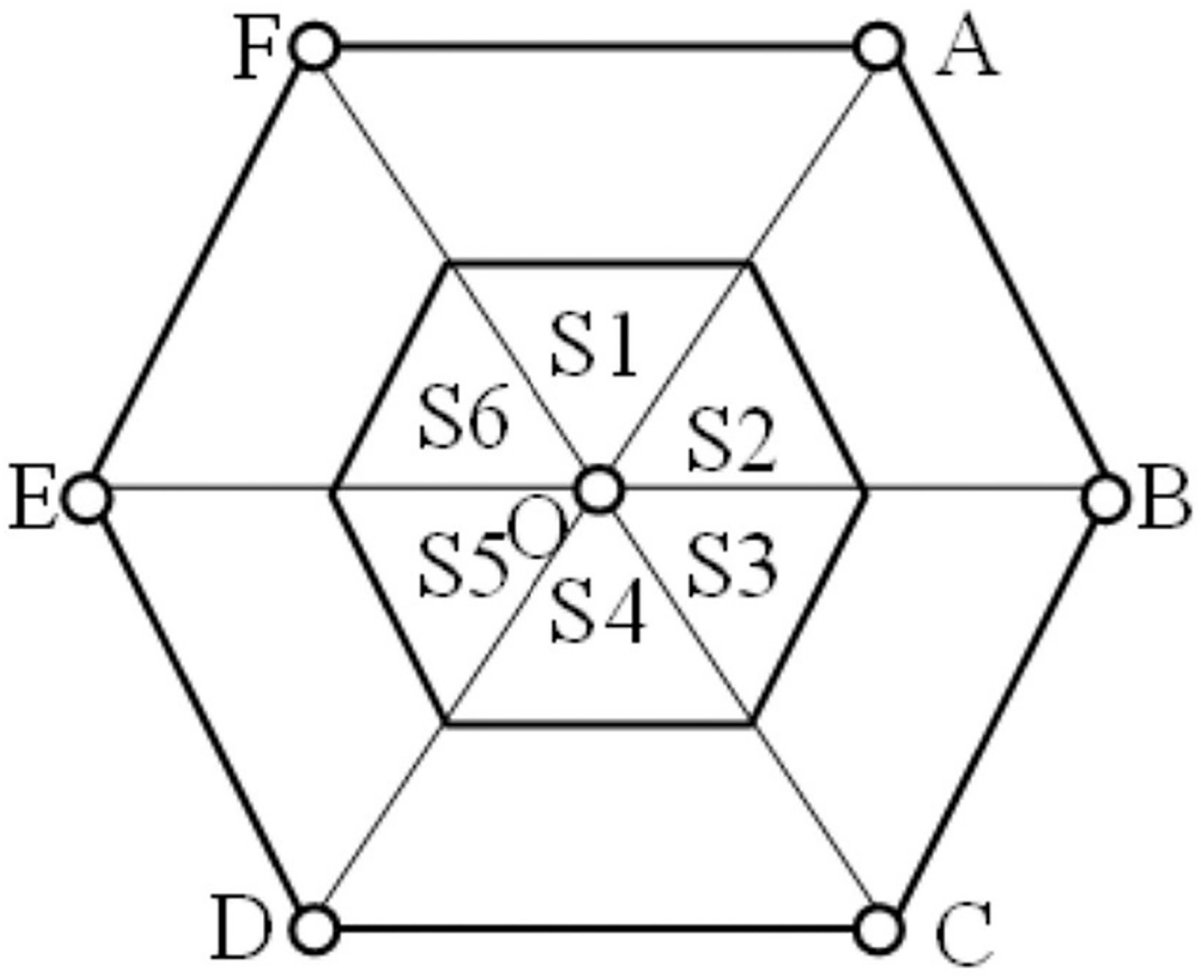
Five BSs selection regional distribution.

For the selection of five base stations, the analysis method is similar to that of four base stations. After analysis, we use five base stations with trapezoidal layout. In a cellular cell, according to the distribution area of target points shown in the Fig 7, the dynamic five base stations selection (DBSS5) is as follows:

*S*_1_ → ▫*EFABO*, *S*_2_ → ▫*FABCO*, [*S*_3_ → ▫*ABCDO*, *S*_4_ → ▫*BCDEO*, *S*_5_ → ▫*CDEFO*, *S*_6_ → ▫*DEFAO*.

where ▫ represents the polygon followed by letters that represent the vertices of the BSs. Based on the above analysis, the dynamic base station selection method (DBSS) is summarized as follows:

1. Determine the initial target location (*x*, *y*) by the initial base station array.
2. Find the nearest base station (*x*_0_, *y*_0_) in the cellular networks by the (*x*, *y*) location (section 2.2.1).
3. Calculate GDOP values of six parallelogram base stations centered on (*x*_0_, *y*_0_) at point (*x*, *y*), and use the base station array with the lowest value as the positioning base station (section 2.2.2).
4. Simplify the calculation of GDOP by dynamically selecting the corresponding positioning base station array according to the rule in section 2.2.3 using the (*x*, *y*) initial location information.

## 3 Simulation experiment

In the experiments, we tested the measurement error using different number of BSs. RMSE (Root Mean Square Error) was selected as the measurement standard. Set (*x*, *y*) be the target location, $\left(\hat{x},\hat{y}\right)$ be the TDOA estimate value, the RMSE can be calculated as:

$$\begin{array}{c} RMSE=\sqrt{{\left(x-\hat{x}\right)}^{2}+{\left(y-\hat{y}\right)}^{2}} \end{array}$$ (41)

Our system is Win10 64bit, CPU is Intel i7–7700k, 64G memory.

### 3.1 TDOA test

**Fig 8:**
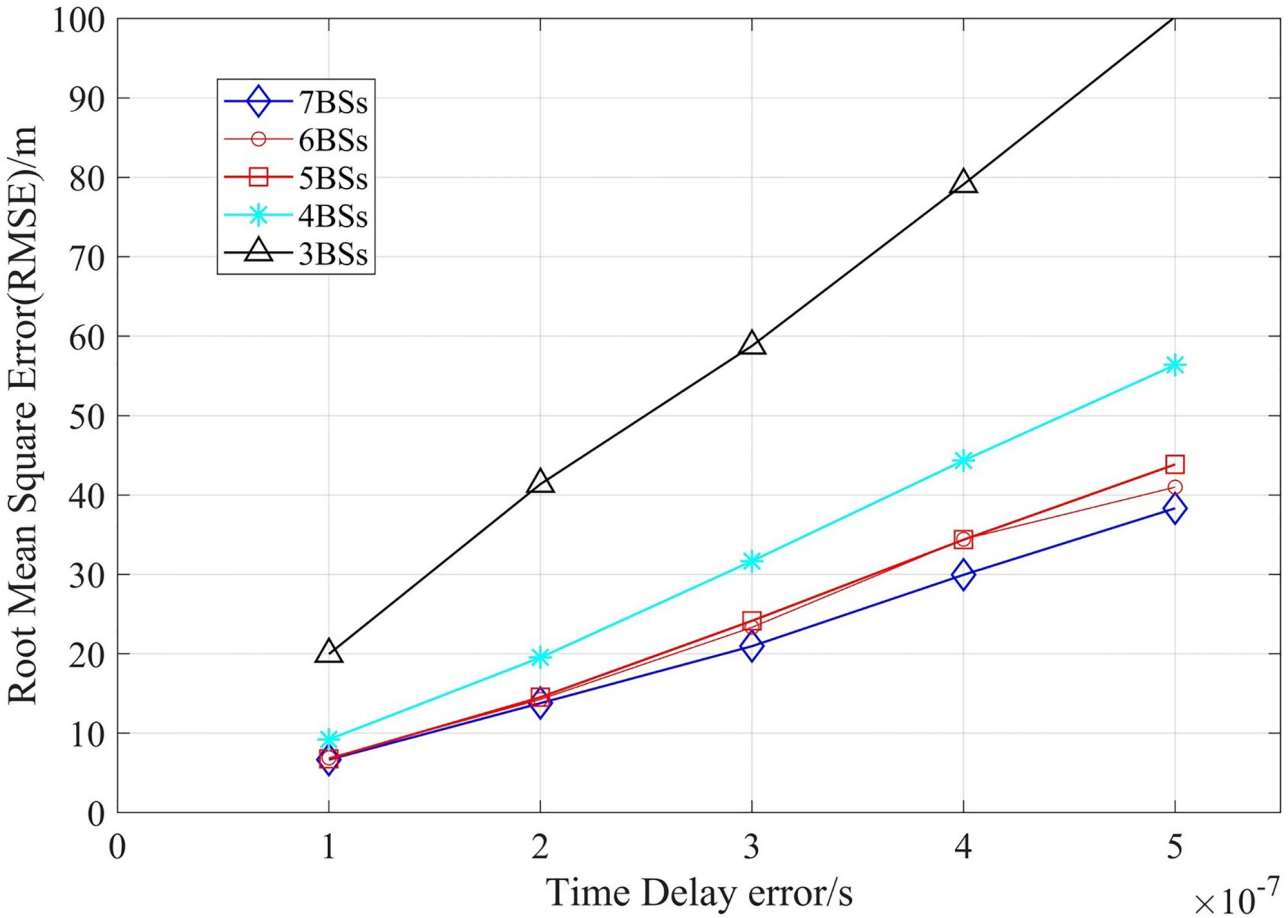
Comparison of positioning accuracy of different number of base stations.

First of all, we use simulation experiments to measure the impact of the number of base stations on the positioning accuracy, and compare the positioning effects of classical TDOA algorithm and Chen algorithm. In this experiment, the mobile cell radius was set as 500m, under different delay errors, the RMSE values measured for different number of base stations are shown in Fig 8.

In Fig 8, when the number of base stations increases, the location RMSE decreases, but when the number of base stations is more than five, the impact of the increasing of the number of BSs on the location error is limited.

**Fig 9:**
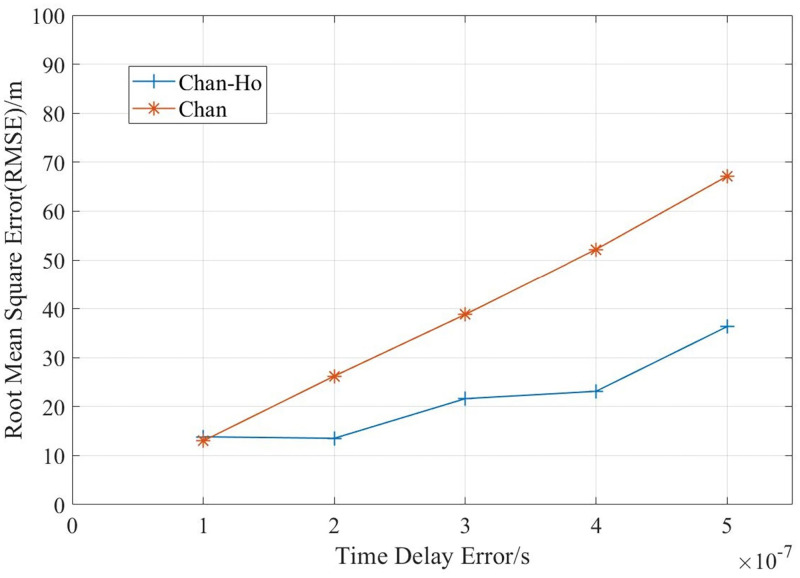
Comparison of Chan and Chan-ho with different delay difference error.

Fig 9 shows the root mean square error of the Chan and Chan-Ho algorithms under different time delay errors. It can be seen that when the time delay error increases, Chan-Ho algorithm is more stable, while the increasing of positioning error is gentle.

### 3.2 Method comparison

In the experiments, we simulated GDOP diagrams using different base station layouts to compare their location accuracy. Set the target transmit power as 30dBm. BS radius of the cellular cell (R) as 500M. Set the measurement error *σ*_ij_ of the base station as the product of the delay error *σ*_*τ*_ and the light speed, which is 0.05m. Delay error *σ*_*τ*_ determined by the SNR of the received signal, was set as 10^—7^s. In a cellular unit with a 1km×1km range, a GDOP value was calculated every 10 meters, and then we drew the contour map.

**Fig 10:**
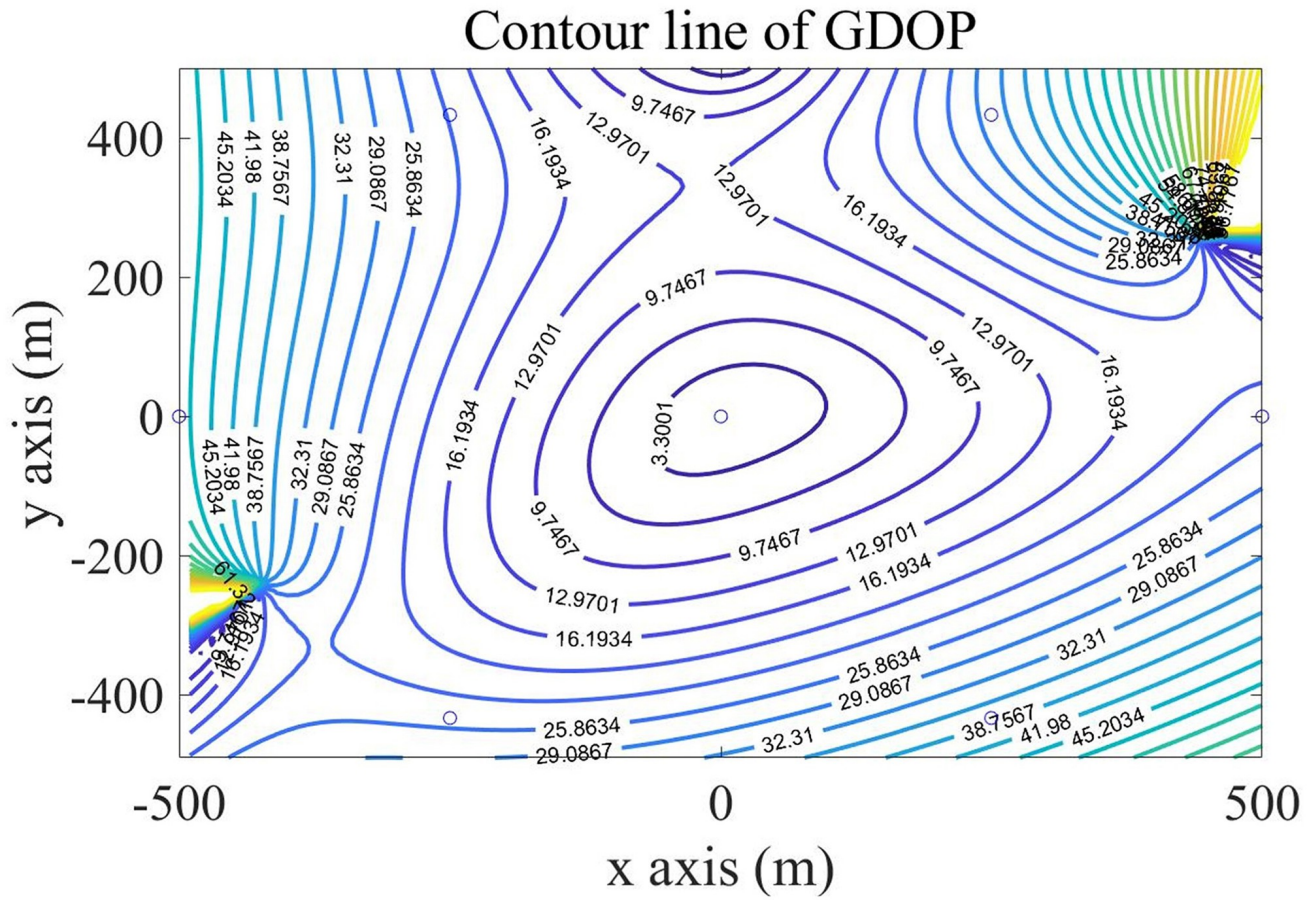
Contour lines of GDOP in parallelogram layout.

According to the above settings, we calculated the GDOP value of different base station selection algorithms in the monitoring area. In order to visually show the GDOP value, we drew the contour map of GDOP value in the monitoring area, Firstly, we drew the GDOP diagram for four fixed base stations. Fig 10 shows the contour lines of GDOP for four fixed BSs. In Fig 10, The horizontal and vertical coordinates represent the scope of the monitoring area and are expressed in meters, the circle represents the location of the base station, the rest is GDOP contour maps, and each contour line is marked with the corresponding GDOP value.

**Fig 11:**
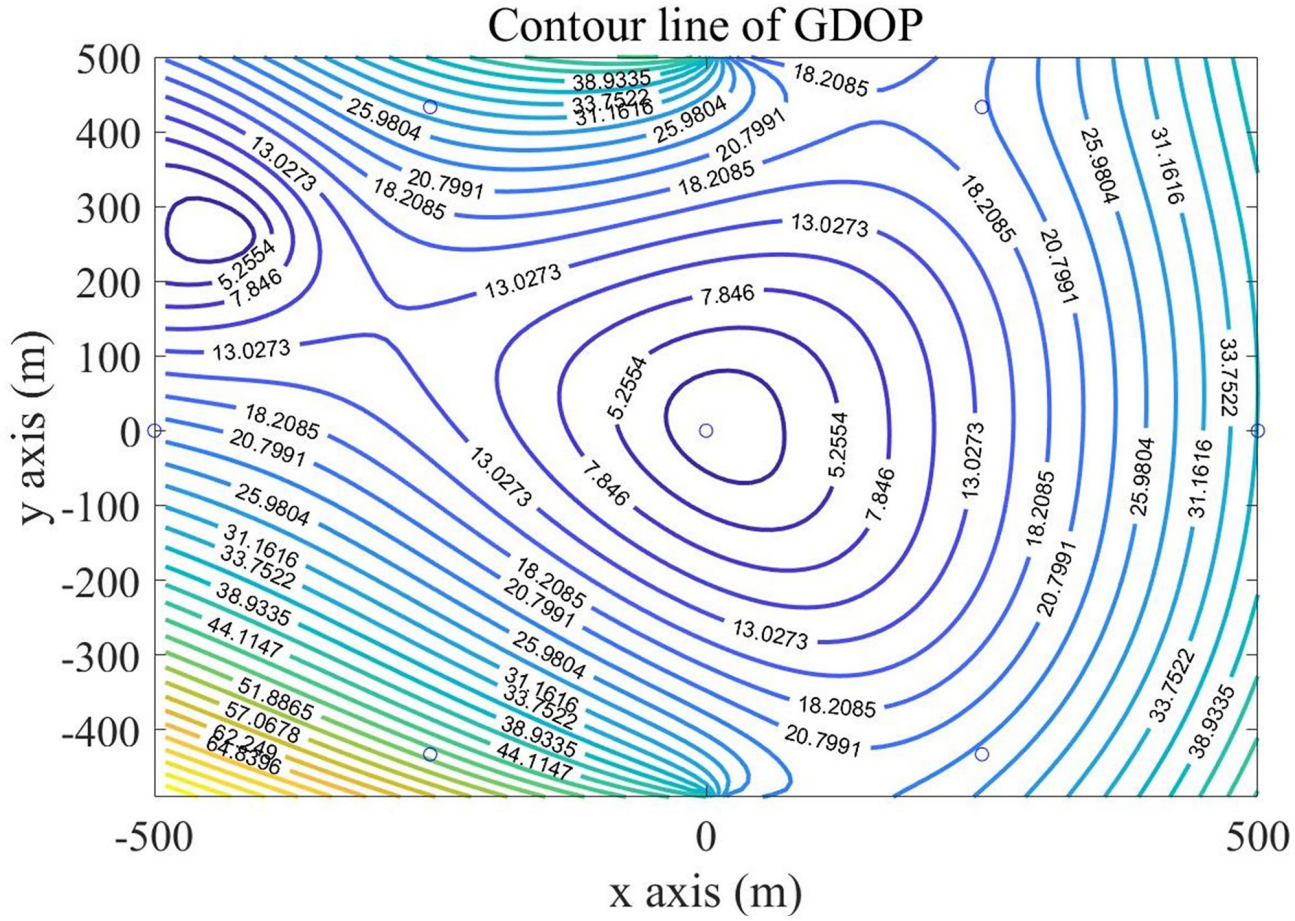
Contour lines of GDOP of four BSs using DBSS4.

**Fig 12:**
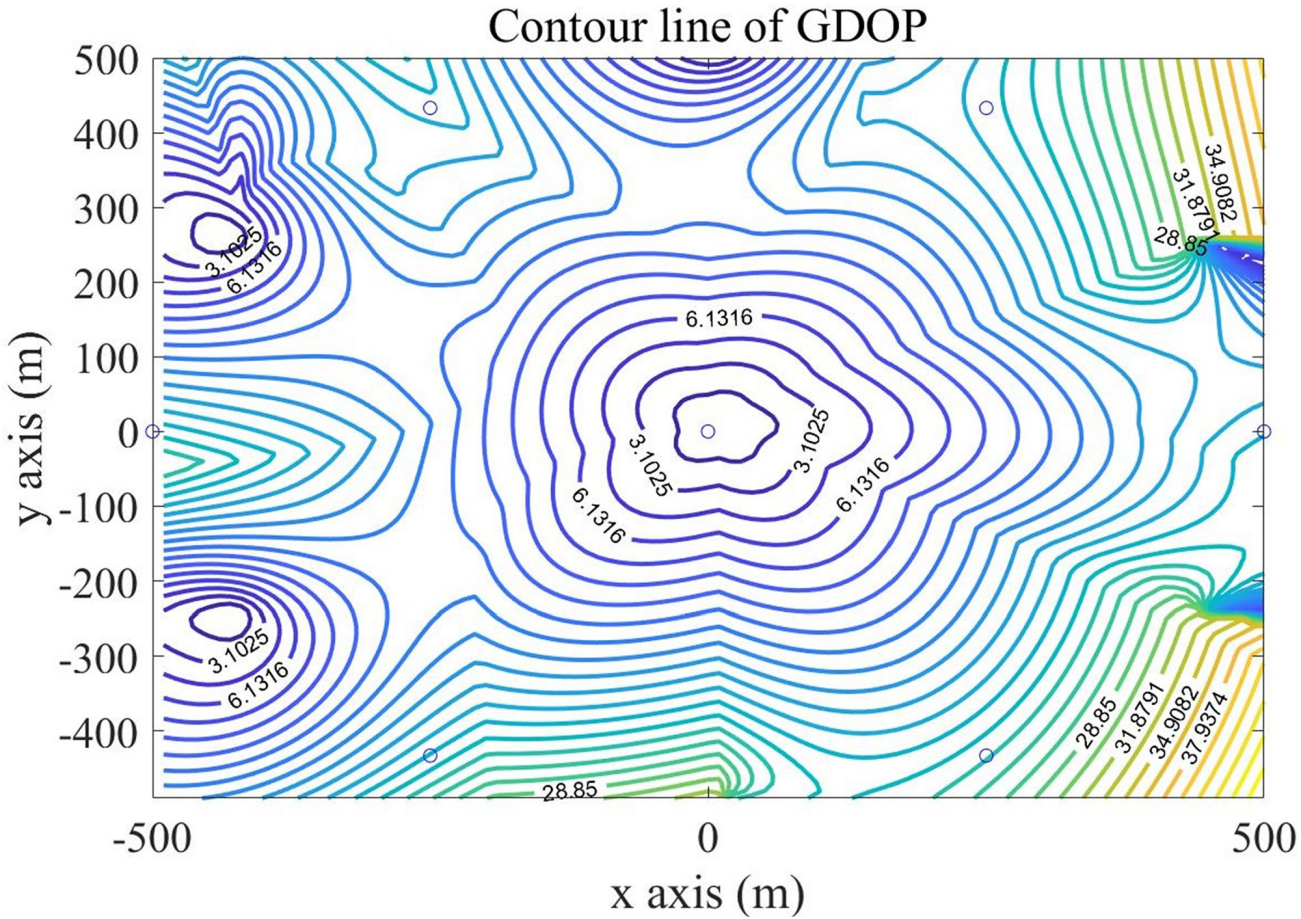
Contour lines of GDOP of five BSs using DBSS5.

Then, using the DBSS method presented in this paper, we calculated the distributions of GDOP contours for four BSs and five BSs, which are shown in Figs 11 and 12 respectively.

For comparison evaluations of our proposed algorithm, we chose two base station selection algorithms for four BSs selection in a cellular cell. One is the Nearly Optimal Sensor Selection method [9] which is named SDR-TDOA. Another is Discrete Monotonic Optimization Based Sensor Selection method [3] which is called POA-AC.

**Fig 13:**
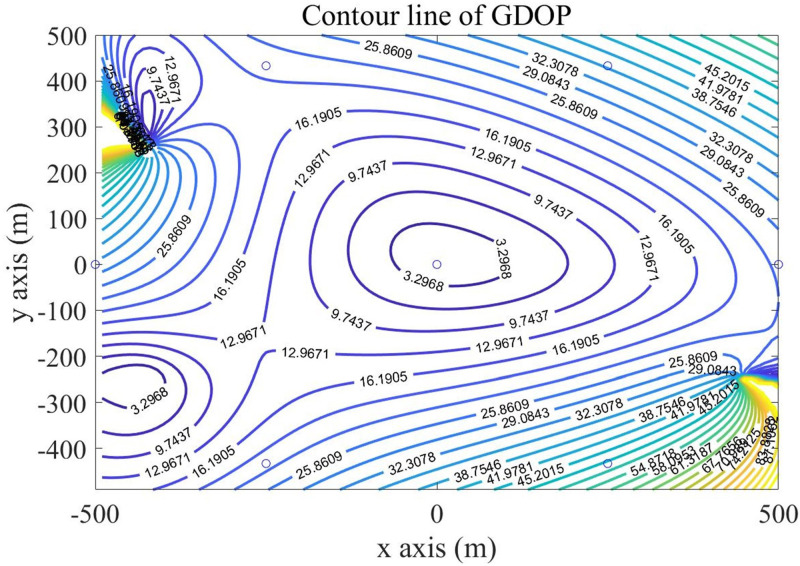
Contour line of GDOP in triangle layout.

Under the same conditions, the BSs selection strategy of NOSS is consistent with the proposed algorithm, so the GDOP diagram of SDR-TDOA is consistent with Fig 11, and the GDOP diagram of POA-AC is showed in Fig 13.

The GDOP contour map gives the distribution map of the positioning error of the algorithm in the monitoring area. Figs 10 to 13 show the GDOP value of different algorithm, from them, we can accumulate value to compare the positioning effects of different algorithms. The accumulated value of GDOP within 250m from the central base station of the cellular unit was used to compare the positioning effect of different BSs selection algorithms. Meanwhile, we recorded the running time, floating points (FLOPs) of operations of each algorithm, and the error ratio reduces compared with the Fixed 4 BSs algorithm. The recorded values are presented in Table 1, in which the running time was recorded by MATLAB under our system configuration, and FLOPs were the estimations of magnitude order rather than accurate values.

**Table 1:** Comparison of various indicators with different method.

Method	Cumulative error (km)	Running time(s)	Error ratio Reduce (%)	FLOPs(10^3^)
Fixed BSs	19.123	0.0412	0	1.2
POA-AC	15.263	0.5284	20.18%	15.3
SDR-TDOA	16.723	1.1935	12.55%	24.2
DBSS4	15.263	0.0486	20.18%	2.1
DBSS5	14.625	0.0521	23.54%	2.5

The computer simulation evaluation and comparison with fixed four BSs layouts in Table 1 showed that the proposed dynamic base station selection (DBSS4) algorithm in mobile cellular networks could reduce more than 20% of the location cumulative error. By comparing with other BSs selection algorithms in Table 1, we can see that POA-AC and DBSS4 have the same choice strategy for four BSs selection, which is better than SDR-TDOA, but POA-AC took more running time and needs to combine with a specific positioning algorithm [7]. DBSS5 had a higher positioning accuracy, but it took more running time and FLOPs than DBSS4.

## 4 Conclusion

For the passive location in in mobile cellular network, the main challenge of BSs selection is that the algorithm is required to be concise and effective, which can quickly update according to the target location, and is independent of the positioning algorithm. In this paper, by analyzing the layout structure of base stations in mobile cellular network and using GDOP as the Optimization objective, we proposed a BSs selection algorithm called DBSS. The novelty of the work is that algorithm only need to calculate the nearest base station of the target, then the location BSs can be dynamically selected quickly. More, the BS selection is independent of the location algorithm. Through the simulation experiment, we discussed as follows:

In the passive location algorithm based on time delay, three base stations can locate the target, but the location accuracy is low. With the increase of the number of base stations, the location accuracy can be effectively improved, but the system overhead will be increased. Moreover, if the number of base stations is more than five, the improvement of location accuracy is limited. Therefore, we give four-base station and five-base station selection algorithms for cellular networks.

In the case of low time delay error, the location error between the typinical TDOA and Chen-Ho algorithm is small, but with the increase of error, Chen-Ho algorithm will perform better, which shows that this algorithm has a strong anti-noise ability.

GDOP value can show the positioning accuracy of each point in different monitoring areas determined by different station layout methods. In this paper, the positioning base station is selected by optimizing the GDOP value. The selection of positioning base station needs to determine the central base station, which is the nearest base station from the target, select the station layout mode, and finally determine the positioning base station in the cellular unit.

In the algorithm comparison, we calculated the GDOP values of DBSS algorithm and three other algorithms, fixed BSs, POA-AC and SDR-TDOA, and provided the contour maps in the monitoring area. By accumulating the GDOP values in the monitoring area, we compared and analyzed the GDOP positioning errors of different algorithms. The results show that our proposed algorithm has the same performance as the exhaustive method in cellular network, and has less floating-point computation, which is more suitable for real-time target monitoring.

In our follow-up research, we will consider the BSs selection for multi-target positioning and moving target tracking, as well as the BSs selection under actual environmental testing conditions, to make the DBSS algorithm more practical in engineering.

The main Notations and Abbreviations in this paper are shown in Table 2.

**Table 2:** A Table of mainly notation and abbreviation in this paper.

Notation	Meaning	Abbreviation	Meaning
*r*_*i*_	Location of ith BS	BS	Base Station
*r*	Location of the target	DBSS	Dynamic Base Stations Selection
*d*_*i*_	the distance from the target to ith BS	TDOA	Time Difference Of Arrival
$\sigma_{ij}^{2}$	the position measurement error between ith and jth BSs	GDOP	Geometric Dilution of Precision
*τ*_*i*0_	time delay of receiving signals from ith BSs to the main station	SNR	Signal-to-Noise Ratio
*σ*_*τ*_	time delay error	RMSE	Root Mean Square Error
*β*	transmission loss of electromagnetic wave	FLOPs	Floating Points per Second

## Supporting information

S1 Data (ZIP)

## References

[1] Chakraborty C. and Rodrigues J.C. A Comprehensive Review on Device-to-Device Communication Paradigm: Trends, Challenges and Applications. ireless Personal Communications. 114(1): p. 185,2020.

[2] Awotunde J.B., Chakraborty C., and Adeniyi A.E. Intrusion Detection in Industrial Internet of Things Network-Based on Deep Learning Model with Rule-Based Feature Selection. Wireless Communications and Mobile Computing. 2021: p. 7154587,2021.

[3] Zhao, Y., et al. Discrete Monotonic Optimization Based Sensor Selection for TDOA Localization. 2019 IEEE Global Communications Conference (GLOBECOM).

[4] Chen, K., et al. An Improved Precise Positioning Method for Intelligent Mine Construction. Journal of Physics: Conference Series. 1566(1): p. 012094 (10pp),2020.

[5] Hussein A.H., et al. A Highly Efficient Spectrum Sensing Approach Based On Antenna Arrays Beamforming. IEEE Access. 8: p. 1,2020.

[6] Wang M., et al. Analysis of the Applicability of Dilution of Precision in the Base Station Configuration Optimization of Ultrawideband Indoor TDOA Positioning System. IEEE Access. PP(99): p. 1,2020.

[7] Mao Z., et al. Moving Source Localization in Passive Sensor Network With Location Uncertainty. IEEE Signal Processing Letters. PP(99): p. 1,2021.

[8] Li K., Ni W., and Dressler F. Continuous Maneuver Control and Data Capture Scheduling of Autonomous Drone in Wireless Sensor Networks. IEEE Transactions on Mobile Computing. PP(99): p. 1,2021.

[9] Dai Z., et al. Nearly Optimal Sensor Selection for TDOA-Based Source Localization in Wireless Sensor Networks. IEEE Transactions on Vehicular Technology. PP(99): p. 1,2020.

[10] Joshi S. and Boyd S. Sensor Selection via Convex Optimization. IEEE Transactions on Signal Processing. 57(2): p. 451,2009.

[11] Chepuri S.P. and Leus G. Sparsity-Promoting Sensor Selection for Non-linear Measurement Models. IEEE Transactions on Signal Processing. 63(3): p. 684,2015.

[12] Ezhilarasan, E. and M. Dinakaran. A Review on mobile technologies: 3G, 4G and 5G. 2017 second international conference on recent trends and challenges in computational models (ICRTCCM).

[13] Singh R.K. and Singh R., 4G LTE cellular technology: network architecture and mobile standards. International Journal of Emerging Research in Management & Technology. 5(12),2016.

[14] Jalal, A. Security architecture for third generation (3G) using GMHS cellular network. in 2007 International Conference on Emerging Technologies.

[15] Ge X., et al. 5G ultra-dense cellular networks. IEEE Wireless Communications. 23(1): p. 72,2016.

[16] Luntovskyy A. and Spillner J. Future Mobile Communication: From 4G To 5G, 5G Enabling Techniques, in Architectural Transformations in Network Services and Distributed Systems. 2017, Springer. p. 211.

[17] Tayyaba, S.K. and M.A. Shah. 5G cellular network integration with SDN: Challenges, issues and beyond. in 2017 International Conference on Communication, Computing and Digital Systems (C-CODE).

[18] Wang L.-C. A new cellular architecture based on an interleaved cluster concept. IEEE Transactions on Vehicular Technology. 48(6): p. 1809,1999.

[19] Sun, R., et al. Station Layout Optimization Genetic Algorithm for Four Stations TDOA Location. in Journal of Physics: Conference Series. of Conference.: IOP Publishing.

[20] Mensing, C. and S. Plass. Positioning algorithms for cellular networks using TDOA. in 2006 IEEE International Conference on Acoustics Speech and Signal Processing Proceedings.

[21] Apkun S., et al. Secure Location Verification with Hidden and Mobile Base Stations. IEEE TRANSACTIONS ON MOBILE COMPUTING. 7(4): p. 470,2008.

[22] Harbi F.S.A. and Helgert H.J. An Improved Chan-Ho Location Algorithm for TDOA Subscriber Position Estimation. International journal of computer science and network security. 10(9): p. p.101,2010.

[23] Laveti, G., et al. Tdoa measurement based gdop analysis for radio source localization. in Procedia Computer Science.

[24] Knapp C. and Carter G. The generalized correlation method for estimation of time delay. IEEE transactions on acoustics, speech, and signal processing. 24(4): p. 320,1976.

[25] Catovic A. and Sahinoglu Z. The Cramer-Rao bounds of hybrid TOA/RSS and TDOA/RSS location estimation schemes. IEEE Communications Letters. 8(10): p. 626,2004.

[26] Shin D.-H. and Sung T.-K. Comparisons of error characteristics between TOA and TDOA positioning. IEEE Transactions on Aerospace and Electronic Systems. 38(1): p. 307,2002.

[27] Cong, L. and W. Zhuang. Non-line-of-sight error mitigation in TDOA mobile location. in GLOBECOM’01. IEEE Global Telecommunications Conference (Cat. No. 01CH37270).

[28] Ishimaru A. Electromagnetic wave propagation, radiation, and scattering: from fundamentals to applications. 2017: John Wiley & Sons.

